# Adolescent Δ^9^-Tetrahydrocannabinol Exposure Selectively Impairs Working Memory but Not Several Other mPFC-Mediated Behaviors

**DOI:** 10.3389/fpsyt.2020.576214

**Published:** 2020-11-12

**Authors:** Han-Ting Chen, Ken Mackie

**Affiliations:** ^1^Department of Psychology and Brain Sciences, Indiana University, Bloomington, IN, United States; ^2^Gill Center, Indiana University, Bloomington, IN, United States

**Keywords:** adolescent, tetrahydrocannabinol, medial prefrontal cortex (mPFC), working memory, cognitive load

## Abstract

As the frequency of cannabis use by 14–16-year-olds increases, it becomes increasingly important to understand the effect of cannabis on the developing central nervous system. Using mice as a model system, we treated adolescent (28 day old) C57BL6/J mice of both sexes for 3 weeks with 3 mg/kg tetrahydrocannabinol (THC). Starting a week after the last treatment, several cognitive behaviors were analyzed. Mice treated with THC as adolescents acquired proficiency in a working memory task more slowly than vehicle-treated mice. Working memory recall in both sexes of THC-treated mice was also deficient during increasing cognitive load compared to vehicle-treated mice. Our adolescent THC treatment did not strongly affect social preference, anxiety behaviors, or decision-making behaviors on the elevated T maze task. In summary, under the conditions of this study, adolescent THC treatment of mice markedly affected the establishment, and persistence of working memory, while having little effect on decision-making, social preference or anxiety behaviors. This study provides further support that adolescent THC affects specific behavioral domains.

## Introduction

In the United States, cannabis is the most commonly used illicit intoxicating substance used by adolescents (1). In 2018, almost 7% of young adults ages 12–17 had used marijuana in the previous month (2). According to the Monitoring the Future Survey, despite the number of marijuana users holding steady at the lowest levels in the past two decades, ~35.7% of 12th graders reported past year use of marijuana, and an increasing percentage of 8 and 10th graders reported daily use (3). Moreover, daily marijuana use continues to outpace daily cigarette use across all grades, reflecting that daily marijuana use has become more popular than daily cigarette use in teenagers (3). While there is a general perception among teenagers that using marijuana is safer than using cigarettes, as will be discussed below, a substantial body of evidence suggests that heavy marijuana users are at risk for developing cognitive deficits. Δ^9^-tetrahydrocannabinol (Δ^9^-THC) is the primary intoxicating compound in marijuana (4). A single dose of 3mg/kg Δ^9^-THC given to adult rats reduces memory performance (5) and multifractality of memory-correlated hippocampus neurons (6). Memory deficits are present in heavy adult human cannabis users after at least 7 days of abstinence, but resolve by 28 days of abstinence (7).

Many clinical and preclinical studies suggest that adolescent exposure to cannabis or cannabinoids may lead to cognitive deficits and emotional instability (8). A common preclinical model of adolescent cannabis exposure has been to chronically treat an adolescent rodent with a cannabinoid, such as Δ^9^-THC or a synthetic cannabimimetic compound such as CP-55940, and then assess adult behaviors. Studies of this design have found that chronic adolescent cannabinoid treatment mimicked the working memory deficit prominent in schizophrenia (9–11), induced defects in object recognition (12, 13), decreased social play and interest in a novel conspecific (12, 14), increased anxiety behaviors (12, 13, 15, 16), and disrupted the decision-making phase of a working memory task (17).

These cognition-related behaviors involve several brain regions, most notably the hippocampus and medial prefrontal cortex (mPFC). Using a Y-maze and rats, the lag of local field potentials between the ventral hippocampus and mPFC indicated that a strong input from the ventral hippocampus to mPFC preceded the correct choice during a working memory task (18). Interestingly, directly applying CP-55940 decreased local field potential oscillations in CA1 and mPFC, also disrupting decision-making demonstrating cannabinoid modulation of the ventral hippocampus to mPFC pathway, suggesting it may be susceptible to chronic Δ^9^-THC treatment (17). A specific effect of Δ^9^-THC on glutamate neurotransmission is suggested by the observation that glutamate levels in mPFC were reduced in young-adult early psychosis cannabis users compared to both non-cannabises using young adults with early psychosis as well as healthy controls. In addition, age and glutamate levels in mPFC were negatively correlated only in cannabis users and only cannabis users had impaired working memory. These findings suggest that cannabis-usage impairs mPFC glutamate levels and working memory (19). Moreover, early adolescent exposure of female rats to a high efficacy CB1R agonist, WIN55212-2, suppressed oscillations in adult mPFC through activation of CB1 receptors (20), and led to chromatin modifications in the prefrontal cortex (16).

Although the enhanced functional activity of the CB1R mediates some adolescent behaviors, such as improved locomotor activity, higher curiosity and impaired anxiety (21), there are conflicting data on sex differences in the effects of adolescent Δ^9^-THC. Several studies suggest that female rodents are more likely to show cognitive deficits following adolescent Δ^9^-THC exposure (15, 16). But other studies have found that there are only slightly differences between sexes in behaviors including open field, elevated plus maze or novel object recognition following adolescent Δ^9^-THC exposure (22). In order to address these open issues, in this study we examined a series of cognition-related behaviors in mice of both sexes after adolescent treatment with Δ^9^-THC to identify consequences of its use on young adult cognitive behaviors and determine if males and females were differentially affected.

## Materials and Methods

### Subjects

C57BL/6J mice (The Jackson Laboratory, Mount Desert Island, Maine, stock # 000664) of both sexes were bred and group housed (2–4 per cage) under constant temperature with a 12-hr light-dark cycle. Food and water were available *ad libitum* except as specified in the experimental design. All experimental protocols were approved by the Indiana University Bloomington Institutional Animal Care and Use Committee.

### Drug Preparation and Administration

Δ^9^-THC (NIDA Drug Supply) was diluted in 100% EtOH to 6 mg/ml stock solution. This stock was then mixed in a ratio of 1:1:18 with Cremophor® EL and sterile normal saline. Vehicle injections consistent of the ethanol, Cremophor® EL and saline in the same 1:1:18 ratio. Daily 3 mg/kg Δ^9^-THC or vehicle intraperitoneal (i.p.) injections (at a volume of 10 ml/kg) were begun at PND28, 1 week after mice were weaned and continued for 3 weeks (until PND49). Both Δ^9^-THC and vehicle mice received 0.39 g/kg of ethanol, a dose devoid of overt behavioral effects. Δ^9^-THC treatment caused male and female mice to gain significantly less weight than vehicle-treated mice over the last few days of treatment (Supplementary Figure 1). Naïve subjects were C57BL/6J mice of a similar age that did not receive daily injections or handling. All mice were housed in the same room in filtertop cages in ventilated racks.

### Behavioral Training and Testing

**Figure 1:**
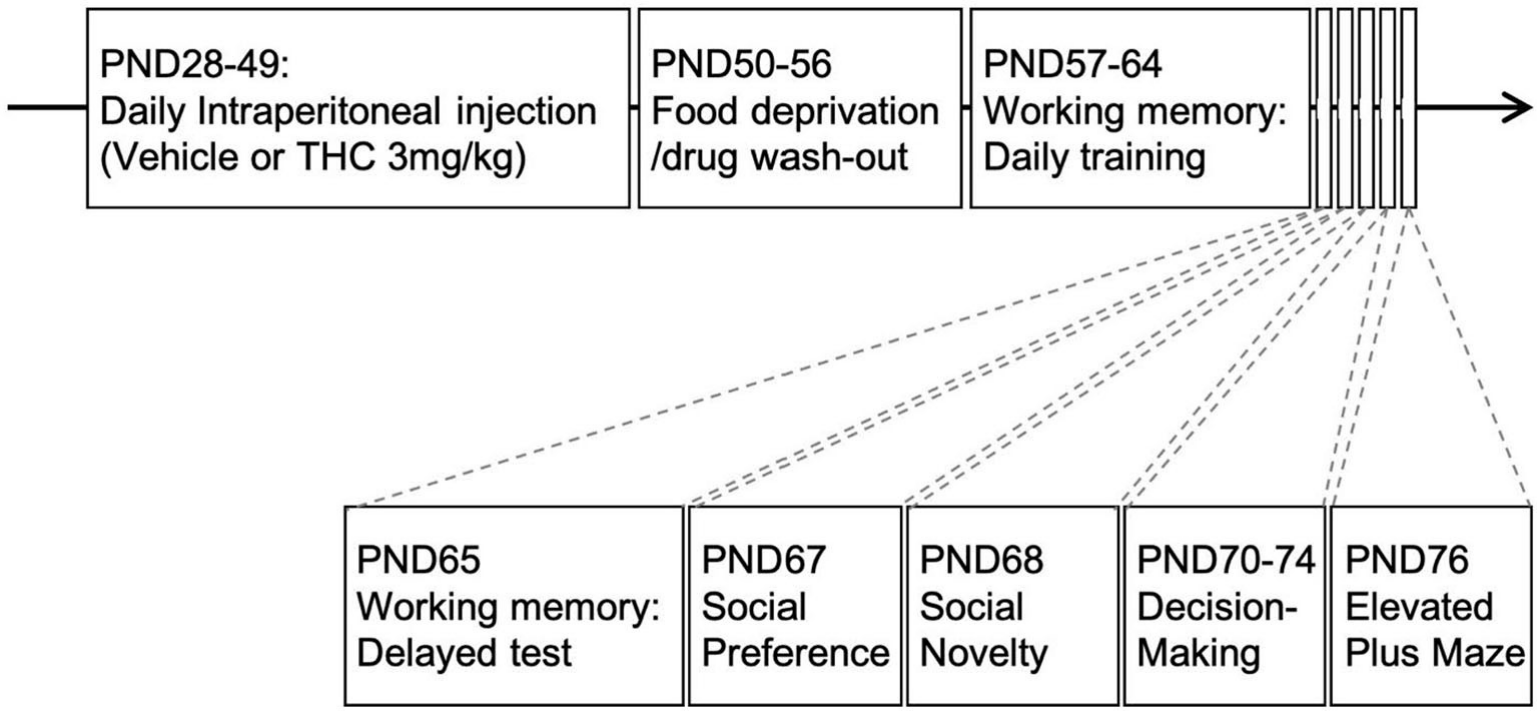
Experimental paradigm. Timeline of experimental design. Adolescent animals received daily intraperitoneal injections of THC (3 mg/kg) 21-days from PND28-49. This was followed by food restriction for 1-week (PND50-56) before behavioral experiments commenced.

Experiments were conducted under reduced illumination (100 lux) and all equipment was cleaned with 75%EtOH and disinfectant solution (Rescue, Oakville, ON, Canada, product #23305) between subjects to reduce the influence of any residual odors on subsequent subjects. Figure 1 shows the overall experimental layout and the order and timing for drug administration and the tests.

### Decision Making

This experiment used a 50-cm elevated T-maze with three 10-cm wide, 30-cm long arms. Sliding doors were set in the side arms and could be removed to mimic an open-arm environment to increase aversiveness for mice approaching the high value reward. The experimental protocol was modified from (23, 24). Animals were first trained to explore and become familiar with the arms of the T-maze for 5 min over 4 days, with one arm having a low reward (one pellet) and the other having a high reward (seven pellets) (Teklad, catalog #2018). On the fifth day, we left the sliding door open on the high reward arm. The location of the high reward was randomly assigned in case animals retained a spatial memory of the high reward location. Mice were allowed to freely explore the T-maze for 5 min during each training session. On the fourth day of habituation (pre-test) and the fifth day (post-test), the animal's movements were both recorded and analyzed by EthovisionXT (Noldus Instruments) to measure the entry frequency and duration of time that each mouse spent in the different compartments of the T-maze. Preference for a zone (arm or center) was calculated as (time in the specific zone)/(time in both arms + center zone)^*^100%.

### Working Memory

A T-maze (each arm 6 × 30 cm) was made from plexiglass with gates at the intersection of each arm that could be closed to force the mouse to turn toward a specific arm. The experimental protocol was modified from (25–27). Briefly, animals were subjected to a daily 4-h food restriction for the week before the training phase, and then had a 2-h food restriction each day prior to testing for the remainder of the experiment. For the first 2 days of habituation, animals were able to freely explore the maze with littermates (day 1) or alone (day 2). For the next 2 days, forced-learning training was conducted with the gate on one-side closed so the mouse could only enter the assigned arm to retrieve a food reward. Delayed alternating response training was started on the fifth day. For this training, 10 trials were conducted each day, with each trial including a forced-choice and a free-choice. The forced-choice trial was conducted identically as the forced-learning training trial (above). After the forced-choice trial, the mouse was trapped in the start arm for 10 s before participating in the free-choice trial. The free-choice trial started with both doors open and the mouse was free to choose either arm, but only the arm opposite from that available during the forced-choice trial contained a reward. After a 1-min delay, the combination of forced and free trials was repeated. Animals' daily success rates were calculated as the percent of the ten trials that were correctly completed to assess their learning ability. The training trials stopped after 8-days of training.

The protocol of increased-delay testing conducted on the ninth day of testing was the same as the training protocol, except we varied the internal delay time during which the mouse was trapped in the start arm from 10 s to 15, 30, and 60 s. Each trial was repeated in blocks of five and the success rate determined for each delay interval as described above.

### Social Behavior

We used a three-chamber test to analyze each mouse's social behavior over 2 consecutive days (The box was 40 × 60-cm and divided into 40 × 20-cm sections with a 6-cm middle chamber in between the two outside chambers). On the first day, social preference was assessed as follows. One wire cup (8-cm diameter) was placed into each of the two outside chambers. To assess social preference, the subject mouse was placed in the middle compartment and allowed to freely explore all three chambers (containing two empty wire cups) for 15 min. After this habituation period, the subject was removed and a familiar mouse (littermate from the same cage as the subject having undergone the same treatment) was placed under one of the wire cups with the other wire cup left empty. The subject mouse was then returned to the chamber and 10 min of video recording performed. Social novelty was assessed on the second day as follows. After a similar 15-min habituation period, the subject mouse was removed. A familiar mouse was placed in one wire cup and a stranger mouse (drug-free, same breed, size and sex, but from another litter) placed in the wire cup in the other chamber. The subject mouse was then returned to the middle chamber and allowed to explore the box for 10 min while its behaviors were recorded. The amount of time each subject mouse spent in the three different compartments and the time spent sniffing the two wire cups were recorded as parameters reflecting the subject's social behaviors using Ethovision XT.

### Elevated Plus Maze

The elevated plus maze was constructed of gray sheet metal with two closed arms and two open arms, each arm was 5 cm wide × 30 cm long. The closed arm had 20-cm vertical walls. The maze was raised 50 cm above the floor. The mouse was placed in the open center area facing in a random direction. The mouse's movements were recorded for 6 min. Distance traveled, entries into each arm and ratio of the time spent in a specific section [(time in open/close arm)/(total time-time in center)^*^100%] as well as the center area were recorded and analyzed by EthovisionXT.

### Statistical Analysis

Behavioral data were analyzed using GraphPad Prism 7 or 8 and are expressed as mean ± SEM. One or two-way ANOVA was used to analyze overall significance for different behavioral experiments, with Sidak's or Tukey's *post-hoc* comparisons to determine significance at specific points. Pearson correlation was used to analyze the correlation between the average training performance of the last 3 days of training and the performance during the delayed memory recall in the working memory test. Three-way ANOVA was used to analyze sex difference for the working memory experiments, with Tukey's *post-hoc* comparisons to determine individual significance at specific days of training or delay time. A *P* < 0.05 was accepted as statistically significant.

## Results

### Adolescent Δ^9^-THC Doesn't Affect Decision Making

**Figure 2:**
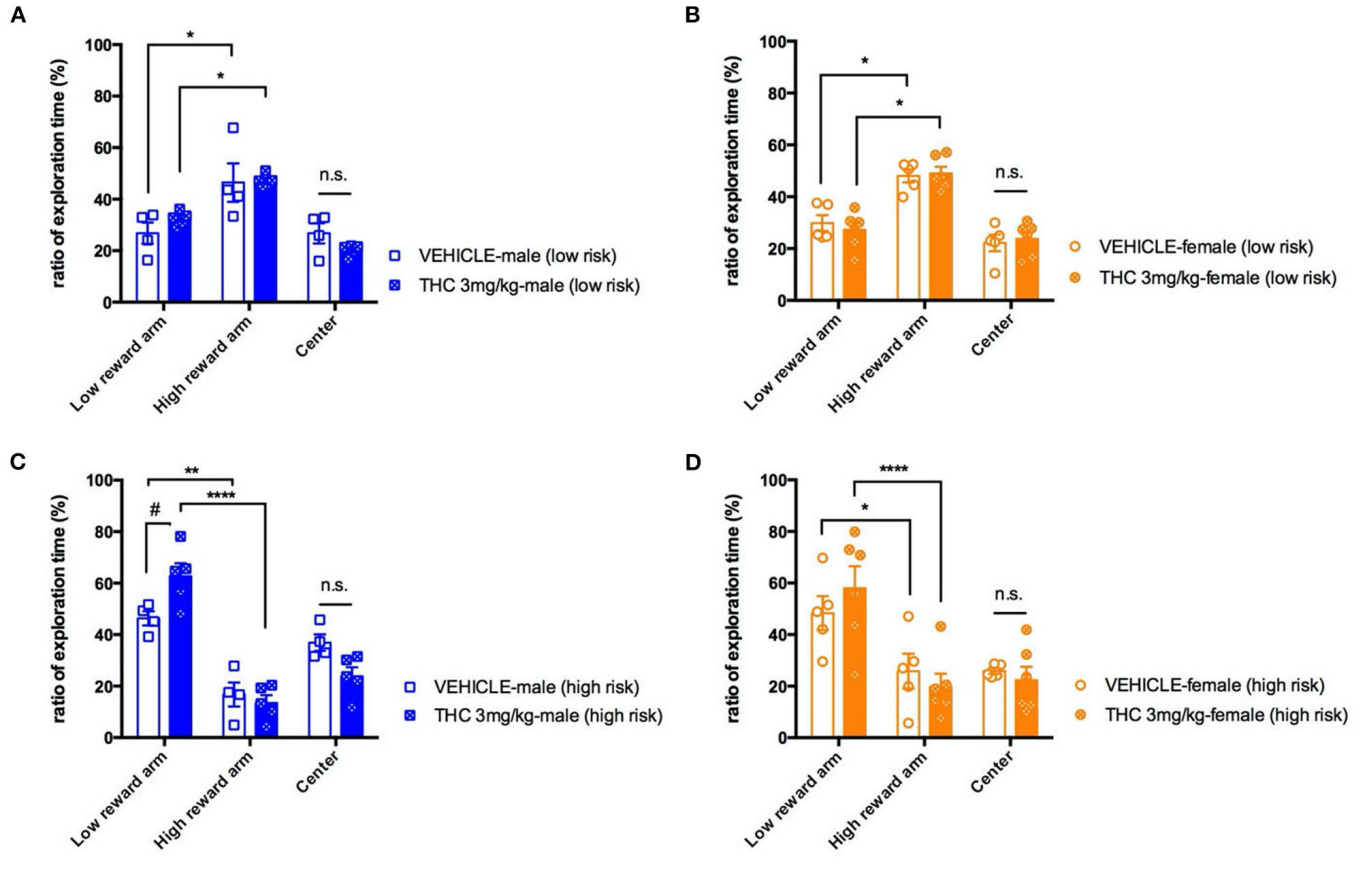
Decision making. Ratio of exploration times spent in different sections of the T-maze on the final day of habituation with both the high- and low-reward arms closed (low risk condition) for male **(A)** and female **(B)** mice. On the next day, mice were placed on the maze with the high-reward arm open, and the ratio of exploration times for male **(C)** and female **(D)** mice for each section were calculated. All data were analyzed by two-way ANOVA with Sidak's test and data are presented as (mean ± SEM). *P < 0.05; **P < 0.01; ****P < 0.001 compared between low and high reward arm. ^#^*P* < 0.05 compared to corresponding vehicle group, n.s., *P* > 0.05 (*N* = 4–5 per group).

Previous studies strongly support that exposing male or female adolescent rats to Δ^9^-THC can induce cognitive impairment in the adults (16). We decided to use an elevated T-maze to examine whether adolescent Δ^9^-THC exposure affects a mouse's decision-making behavior as indicated by the rapidity of decision making under two different levels of risk. Thus, we focused on time in the center area of the T maze (where the decision was being made) when risk increased on the higher reward arm. Mice went through four daily trials of 5-min habituation training with either a high or low number of regular food pellets at the end of each closed arms, and the time spent in the two arms was recorded. By the fourth day of habituation training, both sexes of treated or untreated mice showed an ~2-fold bias toward the high reward arm under the low risk condition [male, F_(2, 6)_ = 18.17, *P* = 0.028; female F_(2, 8)_ = 13.22, *P* = 0.00289] (Figures 2A,B). On the fifth testing day, we removed the walls of the high reward arm to increase perceived risk, which we hypothesized would increase the time spent in the center zone as the decision to enter an arm is being made. Increasing risk significantly increased the fraction of time spent in the center compartment for male mice treated with vehicle [risk, F_(1, 7)_ = 9.145, *P* = 0.0193], but increasing risk did not increase center time for THC-treated male mice. High risk also did not increase the fraction of time female mice spent in the center zone regardless of treatment [risk, F_(1, 9)_ = 0.1360, *P* = 0.7208]. Overall, under a high risk condition, we found there was no significant difference in decision times between the vehicle and THC groups as measured by the fraction of time spent in the center compartment for either males [F_(2, 6)_ = 0.3169, *P* = 0.7399] or females [F_(2, 8)_ = 2.971, *P* = 0.1084] (Figures 2C,D). This suggests that exposure of adolescent mice to 3 weeks of 3 mg/kg Δ^9^-THC doesn't affect this type of decision-making. An interesting secondary outcome of this experiment was that male, but not female, Δ^9^-THC-treated mice had a significant preference for the low reward arm in the high risk situation [male, F_(2, 14)_ = 4.969, *P* = 0.0234; female, F_(2, 18)_ = 0.6295, *P* = 0.5442] compared to vehicle-treated mice.

### Working Memory Acquisition and Consolidation Are Impaired by Adolescent THC

**Figure 3:**
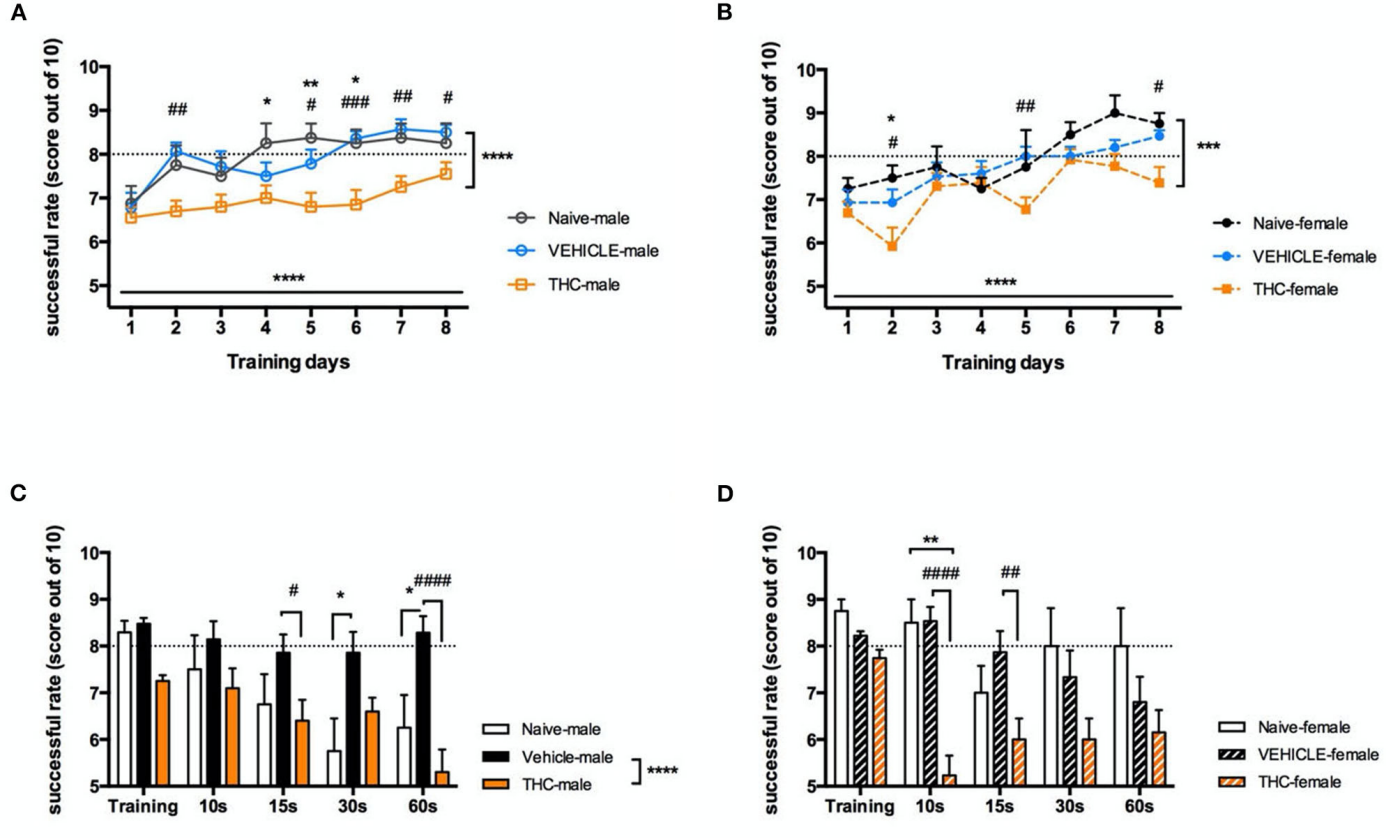
Working memory. Working memory is impaired in adolescent mice chronically treated with THC. Success rate (out of 10 trials) during delayed-alternating T-maze training for 8 days of males **(A)** and females **(B)**. Following 8 days of training, increasingly delayed internal times were tested in 5 trials for male **(C)** and female **(D)** mice. All data were analyzed by two-way ANOVA with Sidak's test and data are presented as (mean ± SEM). *P < 0.05; **P < 0.01 compared between naïve and THC groups. ***P < 0.001; ****P < 0.0001 comparison over time or between vehicle and THC groups. ^#^*P* < 0.05; ^*##*^*P* < 0.01; ^*###*^*P* < 0.001; ^*####*^*P* < 0.0001 compared between vehicle and THC groups (*N* = 4–14 per group).

Working memory is important to retain critical information for a short period, for example, to be retrieved for later use in a multistep task. Here we used the delayed alternating T maze (DAT) to examine whether adolescent treatment with Δ^9^-THC affected working memory. Following 1-week of mild food deprivation and a 4-day habituation period, mice were trained in the DAT. Both male [F_(7, 273)_ = 5.628, *P* < 0.0001] and female [F_(7, 203)_ = 6.785, *P* < 0.0001] Δ^9^-THC- and vehicle-treated mice increased their rate of successful trials over 8 days when subjected to 10 trials/day (Figures 3A,B). Interestingly naïve and vehicle treated mice learned significantly faster than either Δ^9^-THC-treated male [naïve F_(1, 26)_ = 19.5, *P* = 0.0002; vehicle F_(1, 32)_ = 37.87, *P* < 0.0001] or Δ^9^-THC-female [naïve F_(1, 15)_ = 12.29, *P* = 0.0032; vehicle F_(1, 26)_ = 16.68, *P* = 0.0004] mice. The average success rate of both non- Δ^9^-THC treated groups reached 80 percent after 5 days of training, while the average performance of Δ^9^-THC treated mice did not reach this criterion, even after 8 days of training (Figures 3A,B). Next, we investigated whether trained mice could maintain their working memory as the duration of the delay before entering the choice section of the T-maze increased. We chose a 10-s delay as the control to enable comparison with the average value of the last 3 days of training, and the subsequently prolonged the delay to 15, 30, and 60-s. Male vehicle-treated mice were able maintain their working memory for at least 60 s of interval delay [F_(1, 32)_ = 37.90, *P* < 0.0001], however performance by the Δ^9^-THC treated male mice was significantly impaired even after a 15 s delay [F_(1, 20)_ = 12.27, *P* = 0.0022]. Unlike male mice, both naïve and vehicle treated female mice were able keep their working memory at the 80% success rate until an interval delay of 30 s. however THC treated mice were significantly impaired even after a 10 s delay [naïve F_(1, 15)_ = 16.09, *P* = 0.0011; vehicle F_(1, 26)_ = 16.97, *P* = 0.0003] (Figures 3C,D).

To clarify if working memory impairment in the THC-treated mice as delay increased was due to impaired learning of the task, we performed a correlation analysis between the average performance of a mouse during the last 3 training days and its performance during the various delay conditions to determine if the degree of task mastery during training correlated with performance during increasing delay. This analysis found no significant correlation (with Pearson comparison) between average training scores and performance during the working memory task in either sex (For females, average vs. 10 s: *P* = 0.8768; average vs. 15 s: *P* = 0.2886; average vs. 30 s: *P* = 0.2110; average vs. 60 s: *P* = 0.9001. For males, average vs. 10 s: *P* = 0.3168; average vs. 15 s: *P* = 0.1472; average vs. 30 s: *P* = 0.6235; average vs. 60 s: *P* = 0.1357).

To address whether there was a sex difference in either learning the task or recall during increasing delays, we performed a 3-way ANOVA using sex, treatment and training day or delay duration as factors. For daily training, treatment [F_(1, 58)_ = 51.49, *P* < 0.0001] significantly affected performance in the T maze. However, there was no significant effect of sex [F_(1, 58)_ = 0.00004122, *P* = 0.9839] or an interaction between sex and treatment [F_(1, 58)_ = 3.647, *P* = 0.0611]. As for the delayed test, treatment [F_(1, 58)_ = 50.11, *P* < 0.0001] and increasing delay times [F_(3.235, 187.7)_ = 6.394, *P* = 0.0003] had significant effects on performance. However, there was not a significant effect of sex [F_(1, 58)_ = 2.361, *P* = 0.1298] or an interaction between sex and treatment [F_(1, 58)_ = 0.024405, *P* = 0.8773]. Thus, we conclude that sex did not significantly modify the deficit induced by chronic THC treatment of adolescent mice in this task of working memory.

### Adolescent THC Exposure Didn't Affect Social Behavior

Social behavior is an important natural behavior of mice. Here we used a 3-chamber social behavioral task to investigate whether young adult mouse social behavior was affected by adolescent Δ^9^-THC exposure.

**Figure 4:**
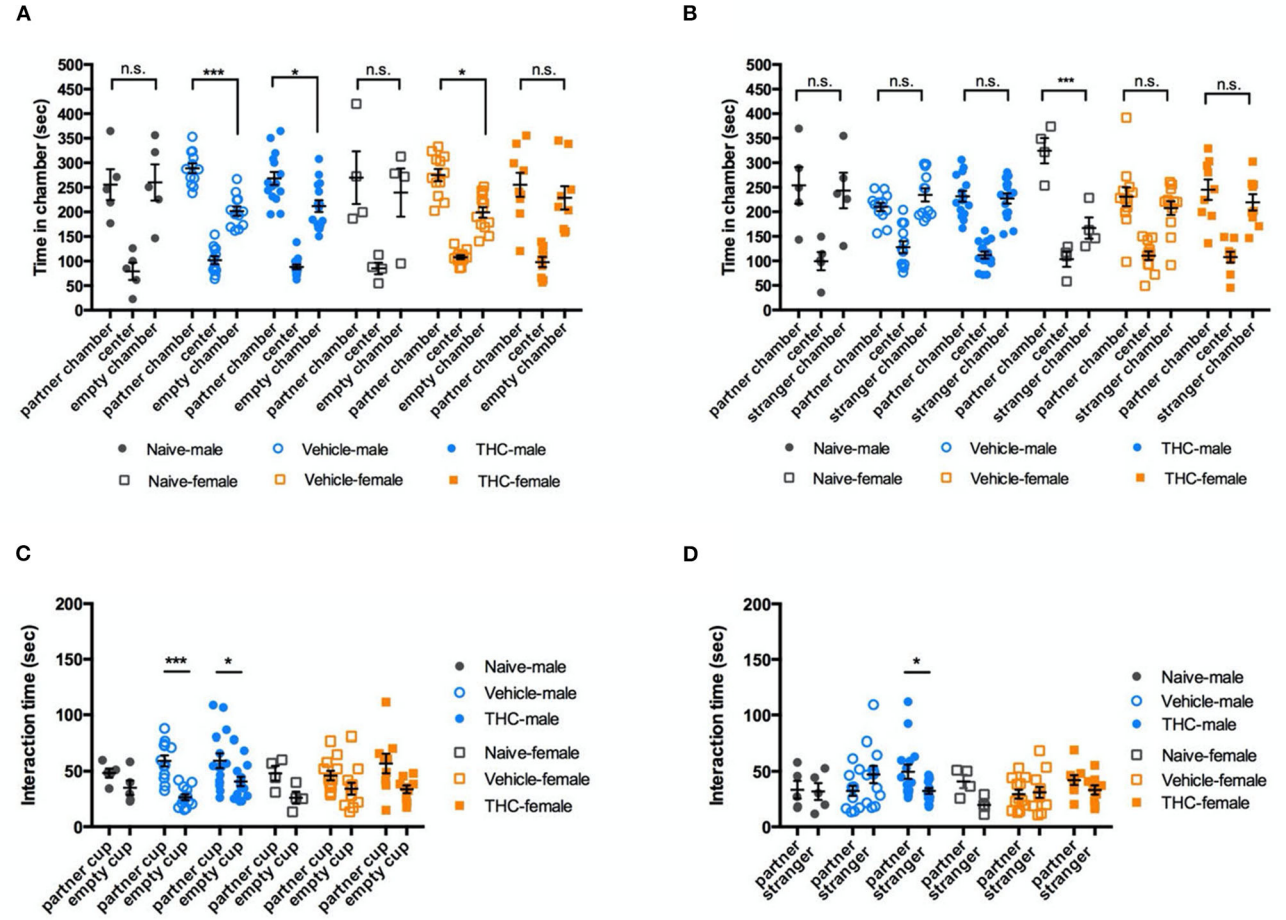
Social ability. Δ^9^-THC treatment did not affect social interactions. Even though male or female naïve, vehicle, and THC treated mice showed different bias in in the chamber paired with an empty wire cup or familiar mouse **(A)** or paired with a familiar mouse or a stranger mouse **(B)**. Similarly, regardless of sex, three groups of treatment caused no difference in the time intending to the chamber **(C)** or the partner vs. stranger cup **(D)**. Data in **(A,B)** were analyzed by two-way ANOVA with Sidak's test, and data in **(C,D)** by repeated-measures two-way ANOVA with Tukey's test. Data are presented as (mean ± SEM). *P < 0.05; ***P < 0.001 compared between partner and empty chamber or *P* compared between familiar and stranger chamber (*N* = 4–14 per group).

To evaluate social preference, the time a mouse spent attentive to a cup with a familiar mouse (cage mate) inside was compared to the time spent attentive to an identical empty cup. During 10-min of free exploration, vehicle and THC-treated male mice showed a significant and similar preference for the chamber with a familiar mouse [F_(2, 58)_ = 87.27, *P* < 0.0001]. However, naïve male mice showed no significant bias between the familiar mouse and the empty cup. For female mice, there was a significant preference for the chamber with familiar mice [F_(2, 44)_ = 36.13, *P* < 0.0001], but no significant difference between vehicle and THC treatments. [F_(2, 22)_ = 0.8670, *P* = 0.4341] (Figure 4A). We used sniffing behaviors (snout within 2 cm of the cup) to identify a mouse's interaction with the cup. During a 10-min exploration period, male mice interacted more with the cup containing the familiar mouse [F_(1, 29)_ = 17.56, *P* = 0.0002], but treatment did not affect this interaction [F_(2, 29)_ = 1.553, *P* = 0.2286]. Female mice also interacted more with the cup containing the familiar mouse [F_(1, 22)_ = 9.757, *P* = 0.0049], and also showed no preference difference between treatments [F_(2, 22)_ = 0.9539, *P* = 0.4118] (Figure 4C).

To examine social novelty, on the next day, we placed a novel mouse (same strain, sex, age, and approximate size but from a different cage) in the previously empty cup as a stranger mouse to examine the tested mouse's preference for social novelty. There was no difference among different treatments (male, [F_(2, 29)_ = 1.447, *P* = 0.2517]; female, F_(2, 22)_ = 1.482, *P* = 0.249) (Figure 4B). Similar to the their social novelty preference, mice showed no bias between interacting with a familiar or stranger mouse [F_(1, 29)_ = 0.07326, *P* = 0.7886; female, F_(1, 22)_ = 4.609, *P* = 0.431], and there was no difference between treatments [male, F_(2, 29)_ = 0.5573, *P* = 0.5788; female, F_(2, 22)_ = 1.680, *P* = 0.2094] (Figure 4D).

In summary, adolescent Δ^9^-THC did not have a major effect on either social preference (Figures 4A,C) or social novelty (Figures 4B,D).

### Effect of Adolescent THC Exposure on the Elevated Plus Maze

**Figure 5:**
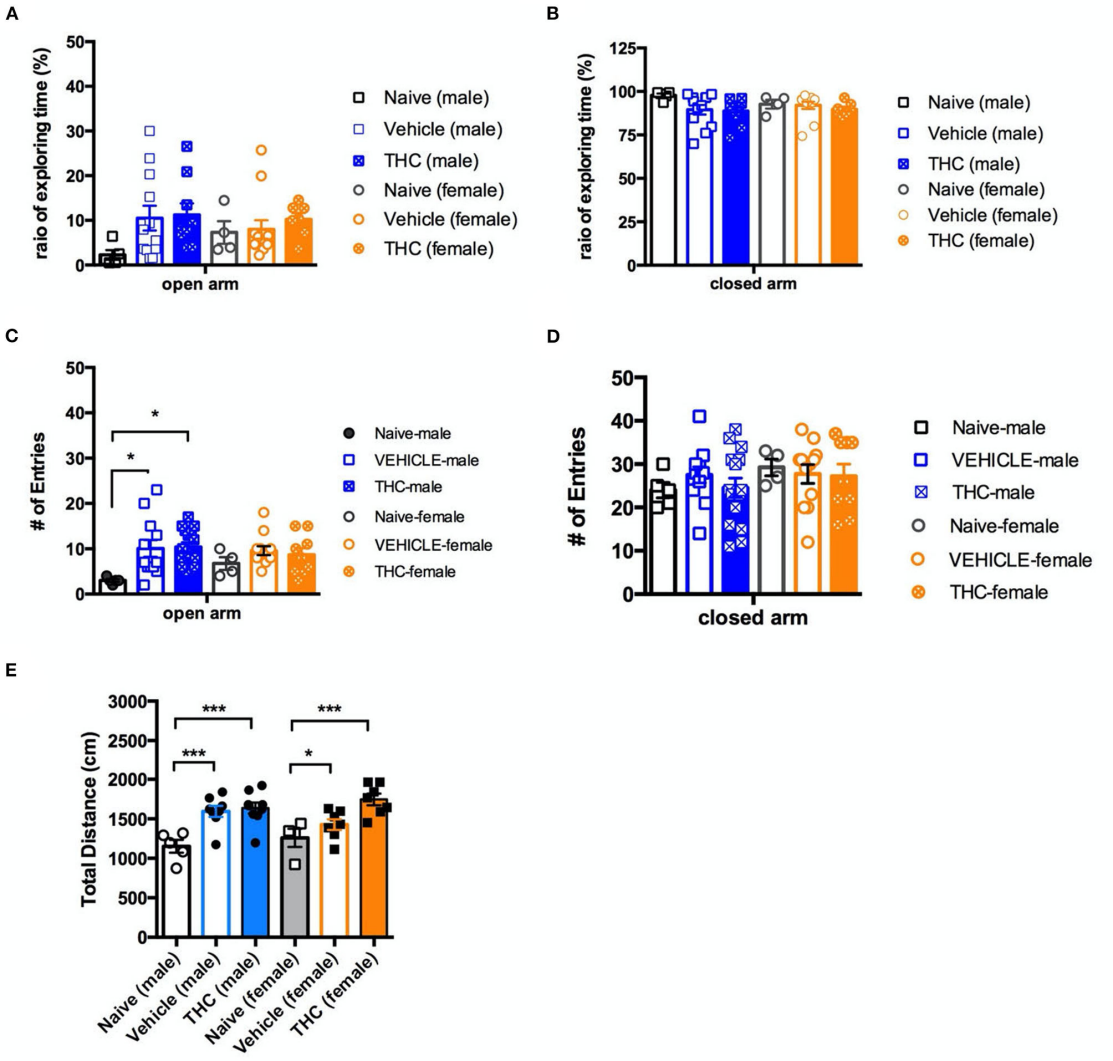
Elevated plus maze. Chronic adolescent Δ^9^-THC treatment doesn't affect mouse anxiety behaviors as measured on the elevated plus maze. Ratio of exploration time **(A,B)** and entries into **(C,D)** the open and closed arms, respectively, of the elevated plus maze as compared to vehicle treated animals. In contrast, naïve mice of both sexes showed less activity as measured by distance traveled, and male naïve mice showed fewer entries into the open arm, as compared to vehicle or Δ^9^-THC-treated mice **(E)**. All data were analyzed by one-way ANOVA with Tukey's test, and data are presented as (mean ± SEM) *p < 0.05; ***p < 0.001 compared to naïve groups (*N* = 4–14 per group).

The last behavior we examined was anxiety as assessed by the elevated plus maze. For male mice on the open arm, there was no significant effect on the ratio of time spent in the open arm by treatment [F_(2, 23)_ = 2.246, *P* = 0.1285] (Figure 5A). However, naïve male mice had significantly fewer entries into the open arm [F_(2, 29)_ = 4.710, *P* = 0.0169] than vehicle or THC-treated mice (Figure 5C). Female mice treated with either vehicle or THC showed no difference on the ratio of time exploring [F_(2, 20)_ = 4.103, *P* = 0.6689] or entries into [F_(2, 20)_ = 0.8757, *P* = 0.4306] the open arm.

Regardless of sex, neither vehicle nor THC treated mice showed a difference on the ratio of time exploring [male, F_(2, 23)_ = 2.246, *P* = 0.1285; female F_(2, 20)_ = 4.103, *P* = 0.6689] (Figure 5B) or entries into [male, F_(2, 29)_ = 0.6787, *P* = 0.5152; female F_(2, 22)_ = 0.1025 *P* = 0.9030] the closed arm (Figure 5D). Vehicle- and THC-treated mice of both sexes were more active (greater total distance traveled) in the elevated plus maze than were the naïve mice, consistent with heightened anxiety [F_(5, 34)_ = 7.802, *P* < 0.0001] (Figure 5E).

## Discussion

### Overall Findings

In this study we demonstrated that administration of Δ^9^-THC to adolescent mice impairs young adult working memory and cognitive behavior, while decision making, anxiety behavior and basic locomotor activity are minimally affected. Interestingly, male Δ^9^-THC-treated mice showed a greater preference for a low reward as risk was increased.

### The Impact of Cannabis Use and the Affected Neurotransmitter/Projections

Several rodent studies show that chronic cannabinoid treatment impairs multiple behaviors, including decision-making (17), social interaction, working memory (12) and novel object recognition (13). However, vulnerability to the adverse effects of cannabinoids may depend on age. For example, cannabinoid treatment of adolescent mice impairs subsequent learning, but a similar treatment of older mice did not (13, 28). These earlier findings identify a specific time window for persistent detrimental effects from adolescent cannabinoids, highlighting the impact of cannabinoids (and likely cannabis) on the developing cerebral cortex, especially the medial prefrontal cortex, a late-developing brain region whose volume decreases dramatically during the teenage years as in undergoes synaptic refinement (29). Our study extends the details of the cognitive behaviors affected by Δ^9^-THC exposure in adolescence.

Evidence implicates the endocannabinoid system and CB1 receptors in impulsive and novelty behaviors, suggesting that decision making behaviors that involve risk assessment might be affected by adolescent THC-exposure (30, 31). However, our results show that, using a risk assessment task vehicle and THC-treated mice spent a similar amount of time considering which arm to choose. A possible explanation for this result is that the amount of risk was insufficient to change behavior. However, this was not the case as both vehicle and Δ^9^-THC-treated mice switched their preference from the high-reward arm to the low-reward arm as risk was increased. Thus, adolescent Δ^9^-THC treatment doesn't appear to affect the speed of the decision-making process occurring while the mice are in the center compartment. An interesting outcome of this study was male (but not female) mice receiving Δ^9^-THC as adolescents had a greater preference for the low reward arm in the high-risk condition compared to male mice receiving vehicle.

Another behavior involving the mPFC and that a number of studies suggest is mediated by glutamatergic synaptic transmission (which others have shown to be affected by adolescent Δ^9^-THC) is T-maze reversal training. In this task, animals first learned the location of a reward in the training phase. Then, the reward is reversed to the opposite side to test the animal's working memory and the learning skill is to identify the reverse task. Accurate learning of this task requires intact glutamatergic transmission in the mPFC. For example, bilateral mPFC administration of either 2.5 or 5.0 μg MK-801 to weanling age rats during the reversal training phase dose-dependently impaired performance (32). Similarly, bilateral administration of Δ^9^-THC into the mPFC also impaired working memory (33). In our study, we found that in mice receiving Δ^9^-THC during adolescence working memory was impaired in the delayed alternating matched T maze. The impairment occurred to a similar extent in both sexes. Our result supports the hypothesis that adolescent Δ^9^-THC treatment could affect the development and thus the adult functioning of the mPFC/glutamate system. Consistent with this hypothesis is a human study that found cannabis use is associated with reduced glutamate levels in the mPFC, which might underlie cognitive impairment (19). Anatomical and physiological experiments have identified projections from the hippocampus (34, 35) and mediodorsal thalamic nucleus (36, 37) to the mPFC. Strong evidence shows that a projection from mediodorsal (MD) thalamus to layer 2/3 of the mPFC mediates the establishment of working memory (36). That working memory is disrupted by adolescent Δ^9^-THC supports the hypothesis that adolescent Δ^9^-THC may affect developing MD-mPFC pathways.

The hippocampus is involved in numerous behaviors such as social recognition memory (38) and spatial working memory (18). Chronic cannabinoid treatment not only induces dysfunction of prefrontal cortical network activity, but it also disrupts interactions between CA1 and the mPFC (17). However, in our study we didn't observe any effect on social interactions in mice receiving chronic adolescent Δ^9^-THC. This result suggests that the pattern and timing of adolescent Δ^9^-THC administration used in the current study has at most a minor effect on this form of hippocampal-influenced memory.

Adolescent THC treatment has also been reported to affect emotional behavior in rodents. For example, female rats receiving adolescent THC showed more immobility in the forced swimming test (FST) and a lower preference for sucrose compared to control female rats, but no differences were seen male rats receiving adolescent THC (15). However, another study in mice found that a short duration (10 mg/kg × 6 injections) adolescent Δ^9^-THC treatment didn't affect the percent of time spent in the open or closed arms for the elevated plus maze (EPM) when examined 4 weeks later (39). Yet another study found that the male adolescent Δ^9^-THC-treatment evokes a delayed anxiety response in adulthood by EPM, which was not observed immediately after drug treatment (13). Here we found no difference between vehicle and Δ^9^-THC -treated adolescent mice in the EPM, in either males or females. This discrepancy may be because of the age of testing [PND52 vs. PND70 (this study)] or strain [CD1 vs. C57BL/6J (this study)]. Together, these varied results suggest that the consequences of adolescent Δ^9^-THC on emotional behaviors may be quite sensitive to cumulative dose, strain and age of the animals.

### Limitations and Future Studies

One notable result of this study is the inconsistency between the naïve and vehicle groups in several tests. Ideally, the naïve group should demonstrate that the vehicle treatment (daily handling + injection of vehicle) does not affect behaviors, however naïve mice had a clear anxiety phenotype on the elevated plus maze, and often behaved differently in other behavioral tests [e.g., delayed recall (Figure 3C) and social interactions (Figure 4A) in males]. Other than not receiving injections and not being handled, naïve mice were treated similarly during adolescence and the behavioral experiments. Considering these factors, the only difference between the naïve and vehicle groups will be handling and injections. Thus, we hypothesize that the handling of the mice for injections acclimates them to removal from cages, handling by the experimenter, etc., making them more accepting of the manipulations involved in behavioral testing. Another possibility is that the low dose of ethanol (~0.4 g/kg), though behaviorally inactive, might have long-lasting effects on behavior through affecting the developing nervous system. Although chronic ethanol intake (e.g., 2 g/kg for 2–3 weeks) during adolescence decreased expression of genes associated with myelin and cholesterol, increased expression of genes related to stress and inflammation, and also expression of 5HT1_A_ (40, 41), the low dose of ethanol encountered in this study is unlikely to affect neuronal development. This could be rigorously tested in future studies by comparing by administration of saline and Cremophor vs. saline, Cremophor, and ethanol to adolescents and then evaluating young adult behaviors (particularly the elevated plus maze).

Another limitation of the study is the small sample size in some experiments, particularly the decision-making study. In this experiment there was no obvious interaction in decision time between the vehicle or THC treated groups (our primary outcome measure), so we ended this experiment after testing a small number of mice. The findings that THC-treated male mice spent a greater fraction of time in the low risk arm or that vehicle-treated male mice spent more time in the center zone could be followed up on in future studies.

The order of behavioral assays was designed with the goal of not conducting behavioral assays using similar apparatus (e.g., DAT and elevated plus maze) consecutively, animals receiving a day of rest between tests, and reducing the overall number of animals used. Since we are investigating the consequence of adolescent THC in young adulthood there is a tradeoff between doing the experiments during young adulthood and giving mice the opportunity to fully recover between tests. Increasing the time between experiments may mean that some experiments will be conducted in animals that are no longer young adults and in some cases the outcomes differ in the different age groups (13). This was also the reason that more stressful tests, for example forced swim or tail suspension tests were not performed. Future studies could follow up on our current results with either more targeted testing (e.g., only social testing) or more stressful tests.

### Final Remarks

Overall, considering our results and those from previous studies adolescent Δ^9^-THC treatment leads to cognitive behavior deficits in both sexes, while social interactions and emotional-related behaviors are less robustly affected.

## Data Availability Statement

The raw data supporting the conclusions of this article will be made available by the authors, without undue reservation.

## Ethics Statement

The animal study was reviewed and approved by Bloomington Institutional Animal Care and Use Committee.

## Author Contributions

H-TC and KM designed experiments, analyzed data, and wrote the paper. H-TC conducted experiments.

## Conflict of Interest

The authors declare that the research was conducted in the absence of any commercial or financial relationships that could be construed as a potential conflict of interest.
