# Supplementary material for: Adolescent Δ9-Tetrahydrocannabinol Exposure Selectively Impairs Working Memory but Not Several Other mPFC-Mediated Behaviors
Source: Front Psychiatry. 2020 Nov 12;11:576214. doi: 10.3389/fpsyt.2020.576214 (PMC7688511; doi:10.3389/fpsyt.2020.576214)
Supplement: Supplementary file 1 [file Data_Sheet_1.PDF]

Supplementary Material

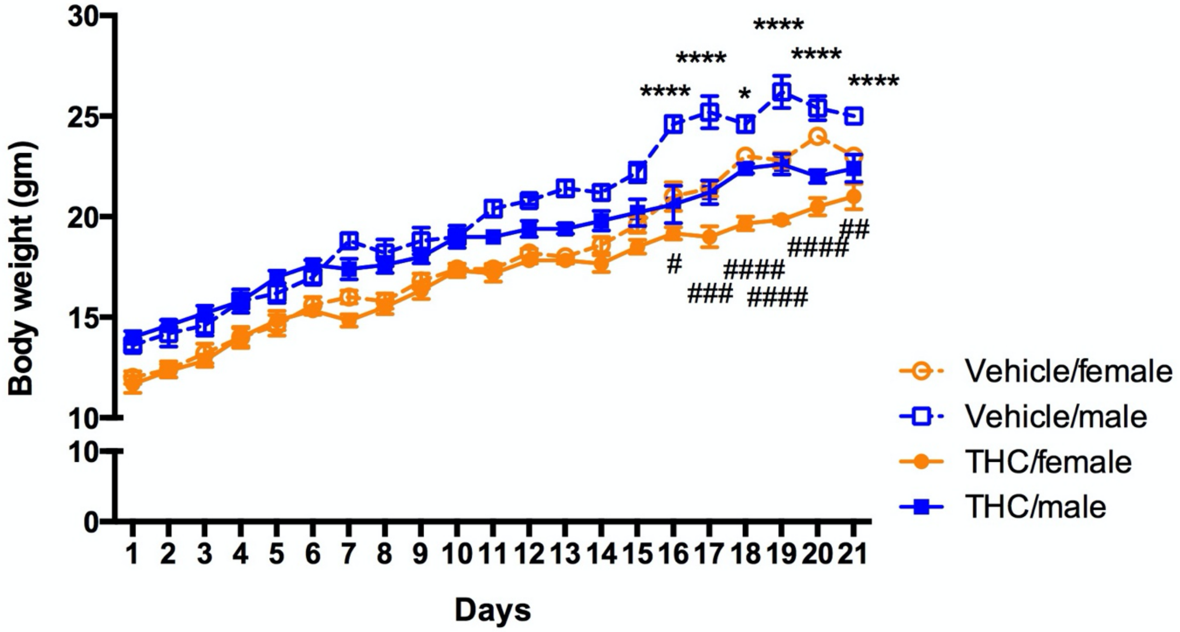

Figure S1. Body weight gain during chronic THC treatment. There are significant interactions for sex, time and treatment [male $F(20, 160)=7.579$, $P<0.0001$; female $F(20, 180)=4.836$, $P<0.0001$], Significant differences for individual days following correction by Sidak's multiple comparison test are shown. For both sexes, body weights were significantly different after 16-days of treatment. Data (mean ± SEM) was analyzed by two-way ANOVA with Sidak's test. *, $P<0.05$; ****, $P<0.0001$, compared with male vehicle treatment; #, $P<0.05$; ##, $p<0.01$; ###, $p<0.001$; ####, $p<0.0001$, compared with female vehicle treatment.

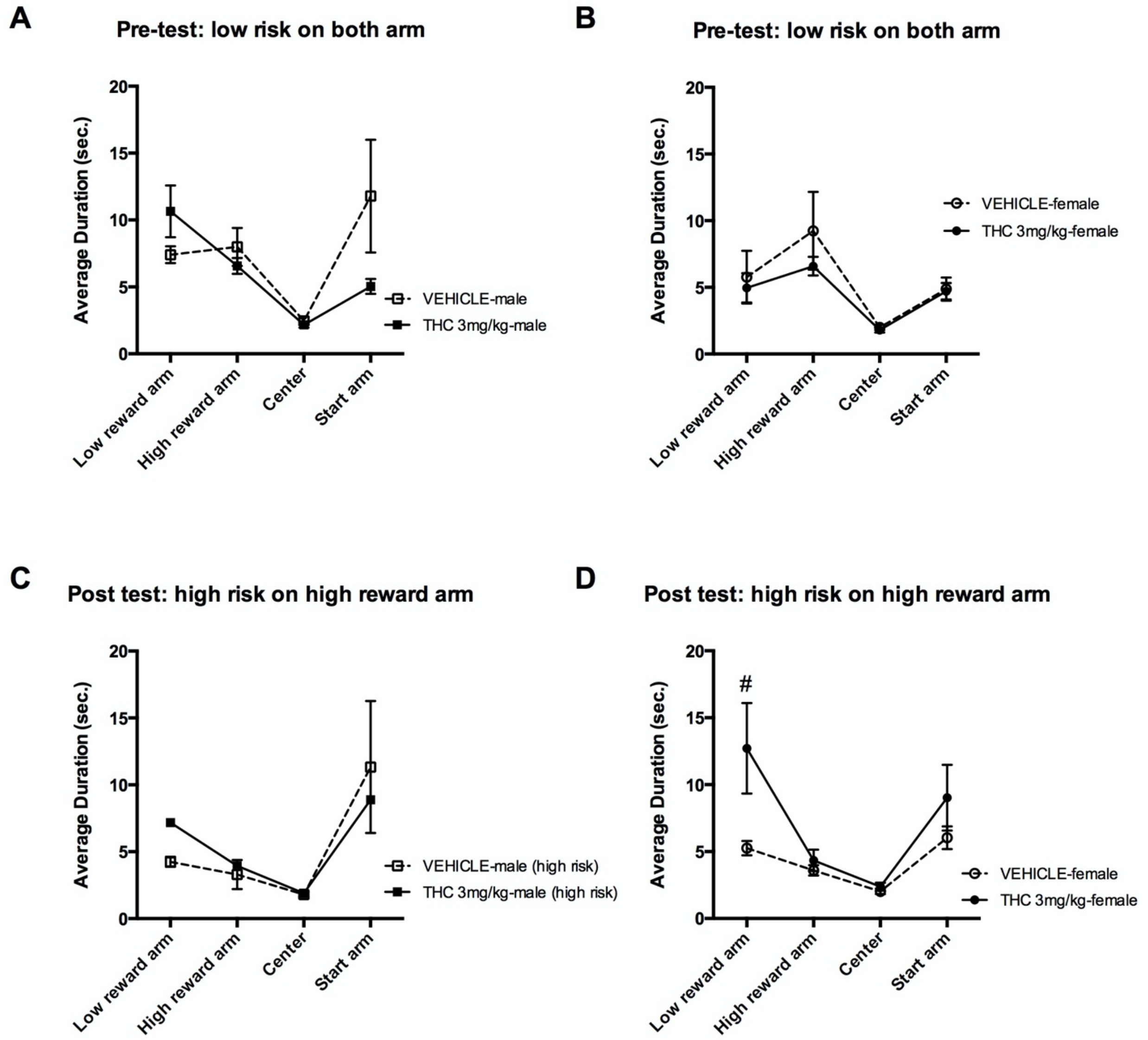

Figure S2. Average time spent in the different sections of the T maze, Comparison between high and low risk condition on vehicle treated (A) and chronic THC treated mice (B). Further comparison between vehicle and chronic THC treated mice on the final habituation day under low risk condition (C) or high-risk condition (D). All data (mean ± SEM) were analyzed by two-way ANOVA with Sidak's test. #, $P<0.05$, compared with female vehicle treatment (N =9-11 per group)

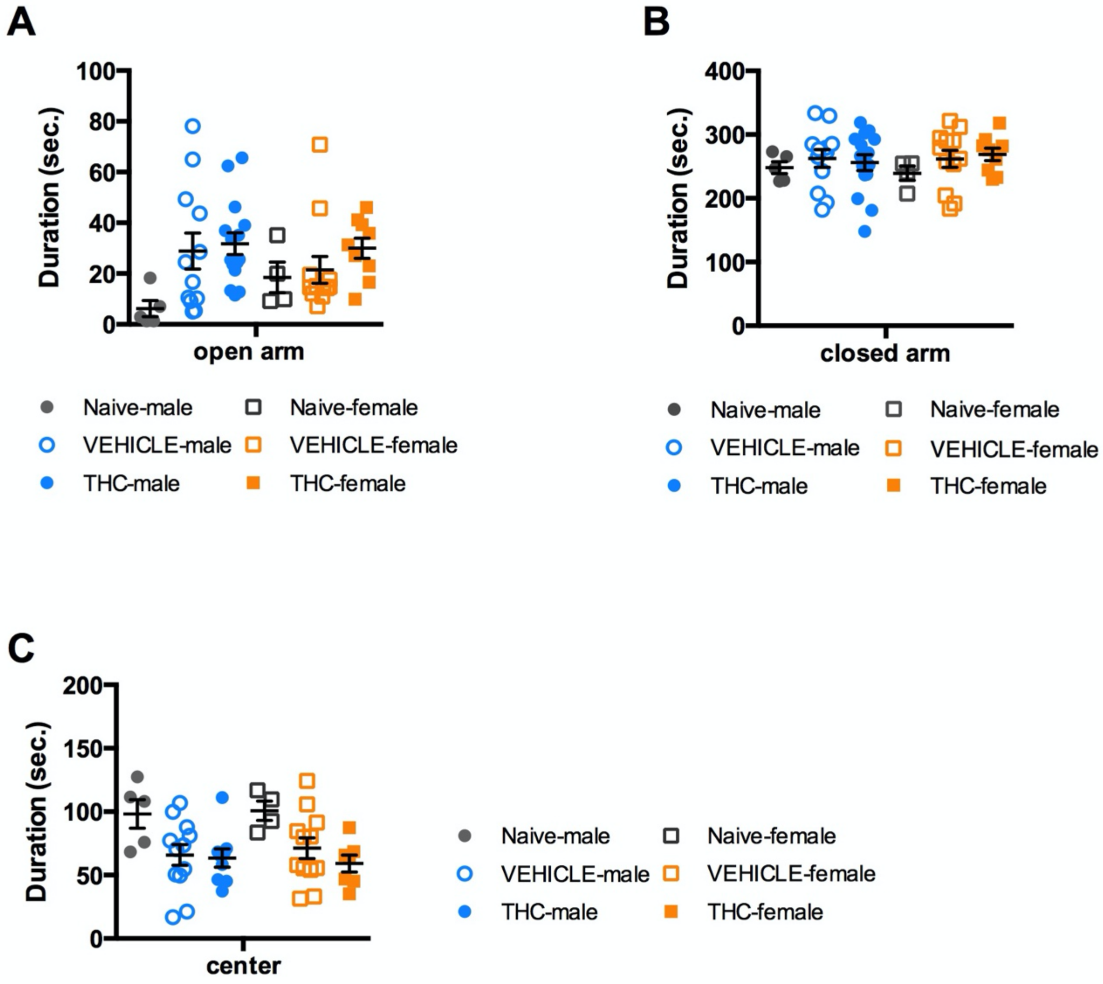

Figure S3. Time mice spent in different areas of the EPM. Time spent by male and female mice in the open (A) and closed arms (B). Time spent in the center zone (C). There were no significant differences between treatments for time spent in the open arm, closed arm or center area, all data were present in (mean ± SEM) and analyzed by one-way ANOVA with Tukey's test.
